# Jellyfish Peptide as an Alternative Source of Antioxidant

**DOI:** 10.3390/antiox12030742

**Published:** 2023-03-17

**Authors:** Lichao Teng, Xueqin Wang, Huahua Yu, Rongfeng Li, Hao Geng, Ronge Xing, Song Liu, Pengcheng Li

**Affiliations:** 1 CAS and Shandong Province Key Laboratory of Experimental Marine Biology, Center for Ocean Mega-Science, Institute of Oceanology, Chinese Academy of Sciences, No. 7 Nanhai Road, Qingdao 266071, China; 2 Laboratory for Marine Drugs and Bioproducts, Pilot National Laboratory for Marine Science and Technology (Qingdao), No. 1 Wenhai Road, Qingdao 266237, China; 3 University of Chinese Academy of Sciences, Beijing 100049, China

**Keywords:** jellyfish, hydrolysis, peptides, antioxidant activity, cytoprotective effect

## Abstract

Jellyfish is a valuable biological resource in marine ecosystems, and blooms been observed in numerous coastal regions. However, their utility is limited by their high water content. Recent research has focused on extracting antioxidants from marine sources. In this study, we obtained jellyfish peptides (JPHT-2) through enzymatic hydrolysis of lyophilized jellyfish powder under optimal conditions and measured their antioxidant activity. Our findings indicate that JPHT-2 possesses significant radical-scavenging activity and reducing power. At a concentration of 0.74 mg/mL, JPHT-2 exhibited a remarkable ability to scavenge hydroxyl radicals, with a rate of up to 50%. The EC_50_ values for scavenging superoxide anion and DPPH radical were 1.55 mg/mL and 1.99 mg/mL, respectively. At the cellular level, JPHT-2 was able to protect HaCaT cells from H_2_O_2_-induced oxidative damage by increasing the level of superoxide dismutase (SOD) in cells. In conclusion, jellyfish peptides with low molecular weight can be easily obtained through hydrolysis with three enzymes and exhibit excellent antioxidant activity and safety. Jellyfish can serve as a promising source of antioxidants.

## 1. Introduction

Jellyfish, a vital constituent of marine plankton, have experienced a population explosion in recent years due to changes in climate and marine environments [1]. This phenomenon has become a global ecological crisis, affecting not only the coastal waters of China but also those of Japan, Korea, the United States, and the Mediterranean coast [2]. The proliferation of jellyfish has had a significant impact on coastal fishery production and maritime activities. Despite their detrimental effects, jellyfish possess nutritional and medicinal properties. As a rich source of protein, it constitutes approximately 50% of its dry weight and contains polysaccharides, iodine, calcium, iron, and phosphorus [3]. Therefore, it is considered a natural health food. With the growing occurrence of jellyfish blooms, there is increasing interest in using jellyfish as a resource. However, its full economic potential has not been realized, and its deep processing requires further attention.

Reactive oxygen species (ROS) are byproducts of normal cell respiration and responses to external stimuli, including superoxide anion, singlet oxygen, hydroxyl radical, peroxide, and nitric oxide, which are highly oxidizing [4]. The concentration of ROS in the body is related to immunity and signal transduction, but excessive accumulation can damage cell membranes and biological macromolecules, leading to tissue and organ damage, abnormal metabolism, and various pathological events [5,6]. The accumulation of ROS has been linked to aging, arthritis, Alzheimer’s disease, cancer, and other illnesses [7]. Imbalance in the oxidation scavenging system can lead to the abnormal accumulation of ROS, and therefore, antioxidants are required to remove such accumulations and maintain the average level of ROS in the body [8]. However, most antioxidants available today are chemically synthesized, such as butylated hydroxyanisole (BHA), butyl hydroxytoluene (BHT), and tertiary butyl hydroquinone (TBHQ). The high levels of chemically synthesized antioxidants can cause organ damage and have mild carcinogenic effects [9]. To mitigate this issue, the search for natural sources of safe antioxidants as substitutes for synthetic antioxidants is gaining more attention [3,10].

Studies have found that compared with proteins, bioactive peptides have various biological activities such as anti-aging [11], anti-hypertension [12], and immune stimulation [13]. Peptides with lower molecular weights are more likely to be absorbed and utilized by the human body through the intestinal barrier to exert their biological functions [14]. The preparation method significantly influences the activity of the final peptide product [15]. Currently, enzymatic hydrolysis is the primary method of peptide production, where proteases are used to destroy the spatial structure of proteins and amide bonds to obtain polypeptides or oligopeptides with different molecular weights, activities, and amino acid compositions [9]. Peptides have found wide-ranging applications in cosmetics, food, medicine, and other fields and play a vital role in regulating bodily functions [16]. Compared to their terrestrial counterparts, marine peptides have several unique characteristics, such as weak antigenicity, good biological compatibility, and low religious barriers. Peptides with antioxidant activity have been prepared and isolated from marine organisms such as fish, shellfish, and their byproducts [17,18,19,20,21,22]. These antioxidant peptides typically consist of 2–20 amino acids and have molecular weights of less than 3000 Da [6,23,24,25]. Smaller-molecular-weight peptides may have more antioxidant activity.

Currently, research on protein resources in jellyfish is mainly focused on jellyfish collagen and collagen peptides. These substances have demonstrated a diverse range of biological activities, including nutritional skin care, immune stimulation, antioxidant properties, antihypertensive effects, and potential as a new biological material. However, the extraction process is complicated and causes a waste of protein [26]. In addition, the molecular weight of most peptides is higher than 1000 Da due to hydrolysis with a single enzyme [27]. This study applied multiple-enzyme hydrolysis to prepare jellyfish peptides with lower molecular weights. This method not only has a higher protein utilization rate but also produces a product that is more easily absorbed by the human body. Furthermore, various tests were conducted to determine the antioxidant activity of jellyfish peptides and to explore their potential as an alternative source of antioxidants.

## 2. Materials and Methods

The salted jellyfish, *Nemopilema nomurai*, has been allowed to use by ethical code, was procured from the Tuandao Market (Qingdao, China), and subsequently subjected to a soaking process with constant water changes every 4 h for four days to eliminate excess salt. Following this, it was transformed into a lyophilized powder to enable enzymatic processing. The 3-(4,5-dimethyl-2-thiazolyl)-2,5-diphenyl-2-H-tetrazolium bromide (MTT), Flavourzyme, Protamex, and Alcalase were acquired from Solarbio Science & Technology Co., Ltd. (Beijing, China). Chemicals such as reduced L-glutathione (GSH), ninhydrin, fructose, potassium ferricyanide, trichloroacetic acid, and ferric chloride were procured from Sinopharm Chemical Reagent Co., Ltd. (Shanghai, China). Other reagents, including 2,2-Diphenyl-1-picrylhydrazyl (DPPH), Nitrotetrazolium Blue chloride (NBT), phenazine methosulfate (PMS), and nicotinamide adenine dinucleotide reduced disodium salt (β-NADH), were purchased from Macklin Biochemical Co., Ltd. (Shanghai, China). HaCaT cells were obtained from China Infrastructure of Cell Line Resource (Beijing, China). Fetal bovine serum (FBS) and MEM culture medium were procured from Hyclone (Logan, UT, USA). Sephadex G-25 was obtained from GE Healthcare (Fairfield, CT, USA). All chemicals utilized in the experiment were of analytical grade.

### 2.1. Preparation of Jellyfish Peptides

#### 2.1.1. Selection of Proteolytic Enzymes

Jellyfish lyophilized powder was weighed and added to ultra-pure water in a 20:1 liquid-to-solid proportion. The ratio of enzyme to substrate was 1500 U/g, and the systems were subjected to a thermostat reaction for a period of 4 h. The proteases were inactivated by placing the hydrolysis systems in boiling water and subsequently allowing them to cool to room temperature. The hydrolysis systems were then centrifuged using a pre-cooled supercentrifuge for a duration of 15 min (4 °C, 18,000× *g*), following which the supernatant was freeze-dried for further analysis. The selection of enzymes and hydrolysis conditions are provided in Table 1, based on the results of ninhydrin colorimetry at 570 nm.

#### 2.1.2. Hydrolyzing with Multiple Enzymes

The lyophilized powder was subjected to a sequential hydrolysis using Alcalase, Protamex, and Flavourzyme enzymes, each operating at 50 °C for a duration of 8 h. Subsequently, the hydrolysates were exposed to boiling water to inactivate the proteases, and the hydrolysates were subjected to centrifugation at 18,000× *g* for 10 min at 4 °C. The supernatant was then collected and, after lyophilization, was used for subsequent antioxidant activity testing.

### 2.2. Determination of Molecular Weight Distribution

The lyophilized powder of jellyfish peptides (JPHT-2) was dissolved in water to create a solution of 10 mg/mL, which was then filtered through a 0.22 µm stream filter membrane and subsequently analyzed with high-performance liquid chromatography (HPLC). The mobile phase utilized a mixture of acetonitrile (85%) and 0.1% trifluoroacetic acid with a flow rate of 0.5 mL/min. Standards including cytochrome c (derived from equine heart), bacitracin, oxidized glutathione, reduced glutathione, and cysteine were utilized. The logarithm of the molecular weight was plotted on the y-axis and the retention time on the x-axis to establish the standard curve for peptide molecular weight. The peak time of JPHT-2 was then used in conjunction with the standard curve to calculate the molecular weight distribution of the peptides.

### 2.3. Antioxidant Activity

#### 2.3.1. Superoxide Anion Scavenging Activity

The methodology for evaluating the scavenging activity of superoxide anions was performed as described by R. Li et al. [5]. To generate the superoxide anions, a Tris-HCl buffer solution (16 mM, pH 8.0) containing 0.5 mL of NBT (300 μM, dissolved in Tris HCl buffer), 0.5 mL of NADH (468 μM, dissolved in Tris HCl buffer), and 1.5 mL of the sample solution was prepared. The reaction was initiated by adding 0.5 mL of PMS (60 μM, dissolved in Tris-HCl buffer) solution to the system. The reaction mixture was then incubated at room temperature for 5 min, following which the absorbance was measured at 560 nm using a universal microplate reader.

Superoxide anion Scavenging effect (%) = [(A_0_ − A_1_)/(A_0_ − A_2_)] × 100 (1)

where A_0_ is the absorbance of the control (without sample), A_1_ is the absorbance of the experimental group with sample, and A_2_ is the absorbance of the blank.

#### 2.3.2. Hydroxyl Radical Scavenging Activity

The hydroxyl anion radical scavenging ability of JPHT-2 was evaluated using a method described by H. H. Yu et al. [28]. Briefly, 1 mL of phosphate buffer solution (150 mM, pH 7.4), 0.5 mL of EDTA-Fe^2+^ solution (220 μM), 1 mL of safranine (0.36 mg/mL, dissolved in PBS), and 1 mL of H_2_O_2_ (3%, dissolved in PBS) were sequentially added to 1 mL of JPHT-2 solution. In the blank group, deionized water was used to replace the sample solution, while in the control group, phosphoric acid buffer was used instead of hydrogen peroxide. The reaction mixture was incubated at 37 °C for 5 min, and the absorbance was measured at 520 nm using a universal microplate reader. The hydroxyl radical scavenging activity was calculated as follows:

Hydroxyl scavenging effect (%) = [(A_1_ − A_2_)/(A_0_ − A_2_)] × 100 (2)

In the formula, A_0_ is the absorbance value of the control group, which uses deionized water to replace hydrogen peroxide; A_1_ is the absorbance of the experimental group containing samples; and A_2_ represents the absorbance of the sample group replaced by deionized water.

#### 2.3.3. DPPH Radical-Scavenging Activity

The DPPH scavenging activity was assessed following the method described by Paul et al. [29]. Briefly, a mixture of 1 mL JPHT-2 solution and 1 mL DPPH solution (0.1 mM in ethanol) was prepared and incubated at room temperature for 30 min in the absence of light. The blank group was prepared by replacing the JPHT-2 solution with deionized water and the DPPH ethanol solution with pure ethanol. The control group was prepared by replacing the sample with deionized water only. The absorbance of the reaction system was measured at 517 nm, and the DPPH scavenging activity of the sample was calculated using the following formula:

DPPH scavenging effect (%) = [1 − (A_1_ − A_2_)/(A_0_ − A_2_)] × 100 (3)

A_0_ is the absorbance value of the control group, which uses deionized water to replace hydrogen peroxide; A_1_ is the absorbance of the experimental group containing samples; A_2_ represents the absorbance of the blank group.

#### 2.3.4. Reducing Power

The reducing power of the sample was determined using the method described by Paul et al. [6,30]. To initiate the reaction, 0.5 mL of the sample was added to 2.5 mL of phosphate buffer (pH = 6.6, 0.2 M) and 2.5 mL of potassium ferricyanide solution (*w/V* 1%), followed by thorough mixing. The reaction mixture was then incubated at 50 °C for 30 min. Subsequently, 2.5 mL of trichloroacetic acid (*w/V* 10%) was added, and the mixture was allowed to stand at 25 °C for 10 min. For analysis, 2.5 mL of the supernatant was transferred and mixed with 2.5 mL of distilled water and 0.5 mL of ferric chloride solution (*w/V* 0.1%). The absorbance of the reaction solution was measured at 700 nm after incubation at room temperature for 10 min. The absorbance of the reaction solution was indicative of the reducing power of various concentrations of JPHT-2 [4].

### 2.4. Protective Effects on Oxidative Damage of HaCaT Cells

#### 2.4.1. Cell Culture

HaCaT cells, an immortalized human keratinocyte line, were obtained from China Infrastructure of Cell Line Resource (Beijing, China). HaCaT cells are commonly used to study the protective effect of natural products against oxidative damage in humans due to their ability to replicate indefinitely while maintaining many of the characteristics of normal human skin cells [22]. Therefore, we chose HaCaT cells to construct the model and cultured them as described by Li et al. [31]. The cells were maintained in MEM-EBSS medium supplemented with 1% penicillin and streptomycin. For optimal growth and proliferation, the medium was supplemented with 10% fetal bovine serum (FBS) sourced from Gibco (Carlsbad, CA, USA). The cells were incubated at 37 °C with atmospheric conditions of 95% air, 5% CO_2_, and 100% humidity.

#### 2.4.2. MTT Cytotoxicity Assay

To evaluate the cytotoxicity of JPHT-2, an MTT assay was employed as described by Li et al. [31]. Briefly, HaCaT cells in the logarithmic growth phase were trypsinized and diluted to form a cell suspension. Next, 100 μL of the cell suspension (1 × 10^3^–1 × 10^4^ cells per well) was added to each well of a 96-well plate and incubated with atmospheric conditions of 37 °C, 95% air, 5% CO_2_, and 100% humidity for 24 h. Subsequently, the medium was replaced with fresh medium containing different concentrations of JPHT-2 for 24 h. To evaluate the cell viability, MTT (0.5 mg/mL) was added to each well, and the cells were incubated for an additional 4 h. After removing the medium, DMSO (150 μL) was added to each well, and the plate was shaken for 10 min in the dark to dissolve the blue-violet formazan crystals produced by the living cells. The absorbance of the resulting solution was measured at 490 nm wavelength. The cell survival rate was calculated using the following formula:

Cell viability(%) = A_1_/A_0_ × 100 (4)

where A_0_ is the absorbance value of the cells in the normal growth group and A_1_ is the absorbance value of the sampling group at 490 nm.

#### 2.4.3. Cell Viability

To induce oxidative damage in cells, a cell suspension was added to a 96-well plate and incubated in a 37 °C incubator for 36 h. Next, the medium in the plate was replaced with fresh medium containing different concentrations of hydrogen peroxide and the cells were further incubated for 12 h. This protocol was used to study the effects of oxidative stress on the cells.

#### 2.4.4. SOD Content in HaCaT Cells

In this study (Section 2.4.3), we investigated the effects of oxidative damage on cultured cells. To obtain cell lysates, we used trypsin to digest and collected cells for lysis. The resulting supernatant was collected by subjecting them to centrifugation at 1000× *g* for 5 min at 4 °C. The SOD activity levels of cell lysates in different groups were determined.

### 2.5. Purification of Antioxidative Peptides

Sephadex G-25 column (1.6 cm × 80 cm) was used to separate the JPHT-2. The constant flow rate of the pump was controlled to be 0.4 mL/min, with purified water as the mobile phase. The elution component was collected with the collector once every 10 min. The absorbance values of the collected components were measured at 215 nm wavelength. The parts with different molecular weights were collected and freeze-driied. Then the superoxide anion scavenging activities and hydroxyl radical scavenging activities of the different peak components were dtermined. The molecular weight distribution of the active fractions was determined using a high-performance liquid chromatography (HPLC) system (Agilent, Santa Clara, CA, USA) equipped with a TSK gel 2000 SWXL column (30 mm × 7.8 mm, Tosoh, Tokyo, Japan).

### 2.6. Statistical Analysis

Data are the mean values of three independent experiments conducted in three replicates. The results are presented with the mean ± standard deviation (SD). Statistical analysis and graphics were performed using GraphPad prism 8. Differences between the means of each group were assessed with one-way ANOVAs followed by Tukey tests at a 95% confidence level. *p*-values less than 0.05 were considered significant.

## 3. Results

### 3.1. Preparation of Jellyfish Peptides with Multiple Enzymes

**Figure 1:**
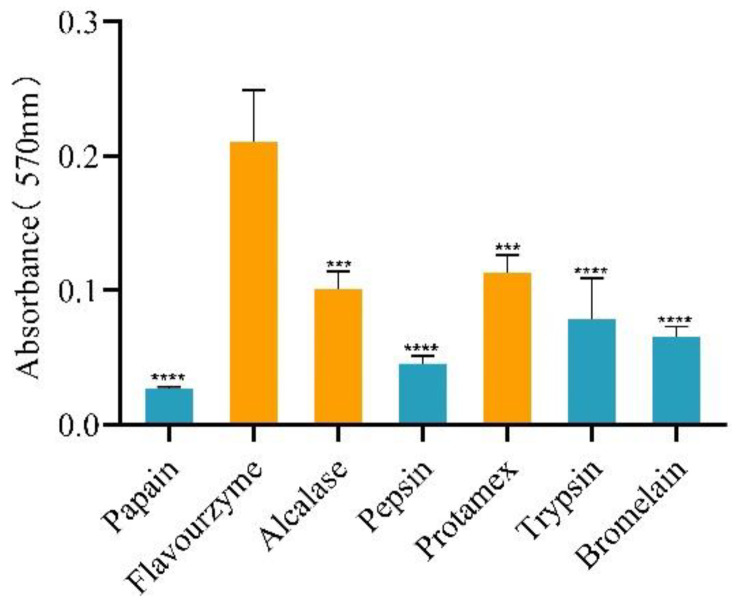
The figure shows the absorbance of seven hydrolysate solutions at 570 nm after reaction with ninhydrin. *** *p* < 0.001 and **** *p* < 0.0001 vs. Flavourzyme. Data are presented as means ± SD.

The activity of a product is determined by the type of enzyme and the method of hydrolysis [2]. Different enzymes cleave substrates at different sites, resulting in peptides with varying activities [32]. Even when the same enzyme is used for hydrolyzing jellyfish powder under different conditions, it can result in products with different molecular weights that can affect the product activity [33]. Moreover, the substrate state is also crucial. Jellyfish homogenate is the substrate of conventional methods for hydrolysis. The enzymic solution obtained with this method has low peptides. As shown in Table 2, the water content of the rehydrated homogenate is much higher than the lyophilized powder, reaching 95.54 ± 0.11%, which is close to the result of N. M. Khong et al. [34]. In general, the high-water content can limit utilization. Therefore, we chose lyophilized powder as the substrate to prepare jellyfish peptides in this study. Figure 1 shows the enzymatic effect of various enzymes on jellyfish lyophilized powder. The peptides react with ninhydrin to form a purple substance with maximum absorption at 570 nm. The content of peptide was positively correlated with the absorbance. The reaction of the hydrolysis solution of Flavourzyme with ninhydrin showed the highest absorbance at 570 nm and was significantly different from that of other components (*p* < 0.0001). Alcalase and Protamex also performed well compared to other enzymes. Flavourzyme, Alcalase, and Protamex have higher hydrolysis efficiency than other enzymes. Therefore, Flavourzyme, Alcalase and Protamex were selected as stepwise hydrolysis enzymes.

**Figure 2:**
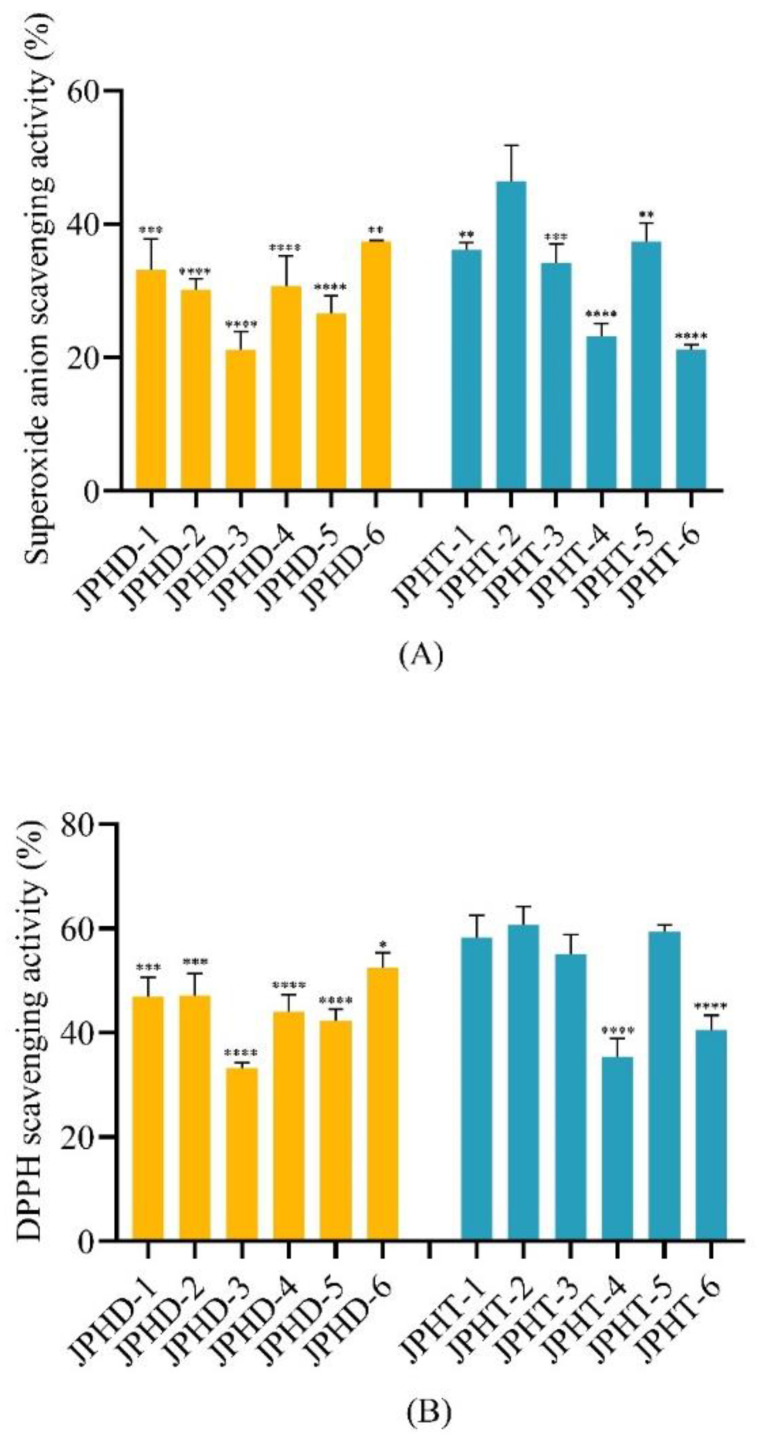
(**A**) Superoxide-anion-scavenging activity of jellyfish peptides obtained by hydrolysis with two or three enzymes. (**B**) DPPH-radical-scavenging activity of jellyfish peptides obtained by hydrolysis with two and three enzymes. JPHD represents the samples obtained with double-enzyme stepwise hydrolysis. JPHT is the hydrolysis product obtained with hydrolysis with three enzymes. * *p* < 0.05, ** *p* < 0.01, *** *p* < 0.001, and **** *p* < 0.0001 vs. JPHT-2. Data are presented as means ± SD.

We used various enzymes to perform hydrolysis on jellyfish lyophilized powder in the order specified in Table 3 and evaluated the hydrolysis effect by assessing their scavenging effects on superoxide anions and DPPH radicals. The result in Figure 2 shows that the scavenging effects on superoxide anions and DPPH radicals of the samples are greatly affected by different hydrolysis orders. Among the two-enzyme hydrolysis samples (JPHD), the sample obtained using Protamex–Flavourzyme (JPHD-6) exhibited the highest scavenging effects on superoxide anions and DPPH radicals. Among the three-enzyme hydrolysis samples (JPHT), the sample (JPHT-2) obtained using Alcalase–Protamex–Flavourzyme showed the highest scavenging effects on superoxide anions and DPPH radicals. This result may be due to the different peptides produced by various hydrolysis sequences. It has been reported that the molecular weight of the hydrolysate is closely related to its antioxidant activity [23]. By comprehensively comparing the antioxidant activities of the products, we find that the superoxide anion scavenging activity and DPPH radical scavenging activity of products obtained using the three enzymes were superior to those of products obtained using two enzymes. The use of three enzymes for hydrolysis enables the substrate to be more comprehensively hydrolyzed, which leads to the release of more small-molecular-weight peptides and contributes to the antioxidant activity of the hydrolysate. Consequently, we selected Alcalase–Protamex–Flavourzyme to prepare jellyfish peptide (JPHT-2), which was subsequently used to determine the molecular weight distribution and antioxidant activity.

### 3.2. Molecular Weight Distribution of JPHT-2

**Figure 3:**
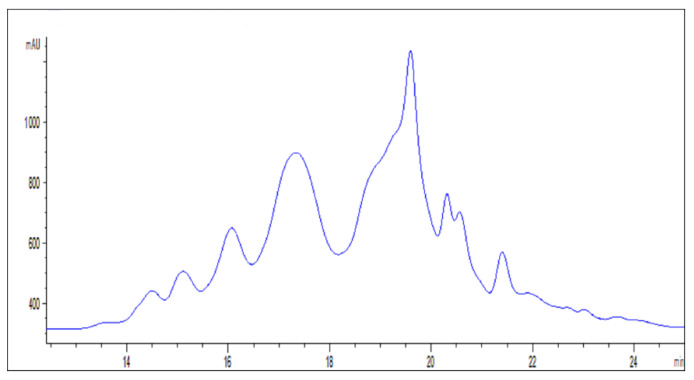
Cellular antioxidant activity treated with different concentrations of JPHT-2.

It is widely recognized that the activity of peptides is influenced by two factors: the amino acid composition of the peptide and the amino acid sequence [9,26]. Peptides possess a more efficient antioxidant activity than individual amino acids due to the diversity of amino acids and particular amino acid sequences [30]. Additionally, the molecular weight of peptides is also affected by their amino acid composition and sequence. Studies have revealed that low-molecular-weight peptides in organisms may have a superior antioxidant activity [4]. They are capable of inhibiting lipid peroxidation and removing radicals, thereby safeguarding the body from oxidative damage. Low-molecular-weight peptides efficiently react with radicals as electron donors, transforming them into stable products, thereby preventing radical chain reactions and safeguarding biological macromolecules such as lipids, DNA, and proteins from ROS attacks [14,35]. The molecular weight distribution of JPHT-2 was determined, as shown in Figure 3 The results in Table 4 show that over 90% of JPHT-2 has a molecular weight below 3000 Da, with 27.39% having a molecular weight between 1000 and 2000 Da and more than 50% having a molecular weight below 1000 Da. The high proportion of low-molecular-weight peptides may be one of the main reasons for the high antioxidant activity of JPHT-2. The use of three enzymes can compensate for the disadvantages of homogeneous cleavage sites, allowing for complete hydrolysis compared to single-enzyme hydrolysis. As a result, more varied small-molecular-weight peptides can be obtained and more varied biological activities of the products are possible. In addition, small-molecular-weight peptides are more stable during gastrointestinal digestion [36]. They can be better absorbed and utilized to perform biological functions in the human body.

### 3.3. Antioxidant Activity

**Figure 4:**
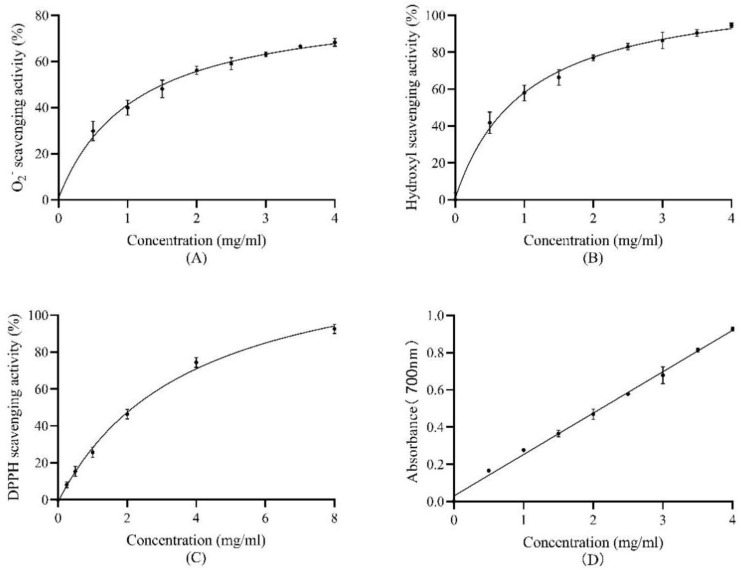
Antioxidant activity of JPHT-2. (**A**) Superoxide anion scavenging activity of JPHT-2 (0–4 mg/mL) obtained by hydrolysis. (**B**) Hydroxyl scavenging activity of JPHT-2 (0–4 mg/mL) samples obtained by hydrolysis with three enzymes. (**C**) DPPH scavenging activity of JPHT-2 (0–8 mg/mL) obtained by hydrolysis by three enzymes. (**D**) Reducing power of JPHT-2 (0–4 mg/mL) samples obtained by hydrolysis with three enzymes. Data are presented as means ± SD.

To further analyze the antioxidant activity of JPHT-2, we evaluated its reducing power and scavenging power against the superoxide anion, hydroxyl radical, and DPPH radical. Glutathione (GSH), a well-known peptide antioxidant with potent reducing power, was used as a control to demonstrate the antioxidant activity of JPHT-2. The experimental results are illustrated in Figure 4A–D.

#### 3.3.1. Superoxide-Anion-Scavenging Activity

In living organisms, oxygen acts as an acceptor for electron transport. When oxygen gains a single electron, it forms a superoxide anion radical. The superoxide anion is the first reactive oxygen species produced by organisms. Its oxidizing ability is weak but it can directly or indirectly form other substances with strong oxidizing properties, such as hydroxyl radicals [5]. Therefore, removing superoxide anions with antioxidants is necessary to protect the body. In the reaction system, the superoxide anion was produced by the oxidation of NADH using NADH–PMS–NBT as the superoxide anion generation system. NBT captures the single electron of the superoxide anion and is reduced to a blue compound that is insoluble in water. The antioxidant can provide electrons for the superoxide anion as electron donors and reduce the absorbance of the system at 560 nm. Therefore, the superoxide-anion-scavenging ability of the antioxidant can be determined [9]. The EC_50_ values represent the JPHT-2 concentration at which the scavenging rate of active oxygen is 50%, used to describe the antioxidant capacity. Figure 4A shows the superoxide anion scavenging ability of JPHT-2 at different concentrations (0.5–4 mg/mL). JPHT-2 has an excellent superoxide-anion-scavenging ability, with an EC_50_ scavenging activity of 1.55 mg/mL. Chi et al. used different enzymes to hydrolyze the skin of bluefin leatherjacket (*N. septentrionalis*) [14]. Alcalase protease-treated hydrolysate was the most active superoxide radical scavenger. The EC_50_ of the hydrolysate is substantially higher than JPHT-2.

#### 3.3.2. Hydroxyl Radical Scavenging Activity

The hydroxyl radical, containing an unpaired electron, is the most active radical and tends to snatch electrons from surrounding molecules to remain stable [35]. In the reaction system, H_2_O_2_ reacts with ferrous ions to produce hydroxyl radicals, which makes the safranine solution oxidize and fade. As shown in Figure 4B, JPHT-2 is very effective in combating the oxidation of hydroxyl radicals to the safranine solution. At a concentration of 1 mg/mL, the hydroxyl radical scavenging activity of the jellyfish protein peptides reaches 57.94%, with an EC_50_ of 0.74 mg/mL. Harada, K et al. determined the antioxidant activity of the freeze-dried giant jellyfish *Nemopilema nomurai* [37]. The results showed the EC_50_ of hydroxyl radical scavenging activity of jellyfish freeze-dried powder was 0.2 g/mL. Compared with this result, the antioxidant activity of JPHT-2 in this study was significantly improved after multi-enzyme hydrolysis. Furthermore, as demonstrated in Table 5, the EC_50_ of JPHT-2 was considerably lower than that of control GSH. These findings support the conclusion that JPHT-2 exhibits a potent hydroxyl radical scavenging activity.

#### 3.3.3. DPPH Radical-Scavenging Activity

DPPH, a nitrogen central radical, is widely utilized to evaluate the antioxidant properties of compounds that function as hydrogen donors [6]. When dissolved in ethanol, the DPPH radical solution appears purple, with maximum absorption at 517 nm. The presence of a radical scavenger leads to the neutralization of the single electron of DPPH, resulting in a lighter system color and reduced absorbance value at 517 nm [30,38]. As indicated in Figure 4C, the DPPH scavenging activity of jellyfish protein peptides was concentration-dependent, with an EC_50_ of 1.99 mg/mL. The antioxidant potency of the jellyfish peptides obtained in this study was found to be superior to that of the crude component extracted from sea cucumber viscera [19], albeit inferior to the positive control GSH.

#### 3.3.4. Reducing Power

The reducing power of antioxidants is determined by their ability to donate electrons. Potassium ferricyanide K_3_[Fe(CN)_6_] in this experiment contains ferric ion, which is oxidizing and has a strong ability to obtain electrons. At this time, JPHT-2 in the reaction system can provide electrons to the trivalent ion and reduce it to potassium hexacyanoferrate K_4_[Fe(CN)_6_]. Potassium ferricyanide continues to react with the potassium hexacyanoferrate to form Prussian Blue (Fe_4_[Fe(CN)_6_]_3_), which has a maximum absorption at 700 nm. As shown in Figure 4D, the reducing power of JPHT-2 increased with the concentration. JPHT-2 exhibits an excellent electron-donating ability, which enables it to supply electrons to radicals, thus enhancing its radical-scavenging activity.

The high antioxidant activity of JPHT-2 can be attributed to their excellent ability to donate electrons, which allows them to scavenge radicals effectively. The amino acid composition is a crucial factor that influences the electron-donating ability of jellyfish peptides [19]. The jellyfish *Nemopilema nomurai* are rich in Gly, Glu, Thr, Pro, Leu, Ala, Lle, Cys, etc. [34]. Previous studies have suggested that mercaptoamino acids, aromatic amino acids, and hydrophobic amino acids play a vital role in determining the antioxidant activity of peptides [23]. The mercaptoamino acid Cys and aromatic amino acids are prone to oxidation, which makes them efficient in protecting the body against oxidative damage [23]. During lipid peroxidation, hydrophobic amino acids, such as Pro, Leu, Ala, and Ile, can combine with unsaturated fats, thereby reducing the content of free radicals and reactive oxygen species generated, hence their potent superoxide anion and hydroxyl radical scavenging ability [27]. Significantly, JPHT-2 exhibits a much higher hydroxyl radical scavenging activity than the control GSH.

### 3.4. Cytotoxicity Effect of JPHT-2 and H_2_O_2_ on HaCaT Cells

**Figure 5:**
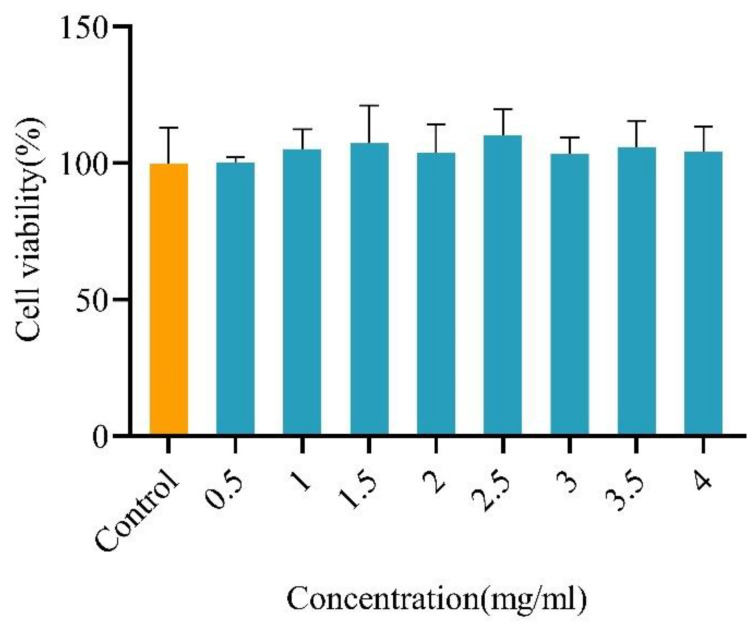
Effects of JPHT-2 on the viability of HaCaT cells treated with different concentrations of JPHT-2 (0.5–4 mg/mL) for 24 h. There was no significant difference between the groups. Data are presented as means ± SD.

Due to the mineral fraction in the substrate and the purity of the protease used in the process of hydrolysis, there are inevitably salt components in the hydrolysate. Thus, JPHT-2 may be cytotoxic and impact the growth of cells [2]. To investigate this, the impact of JPHT-2 on cell viability was measured across various concentrations ranging from 0.5 mg/mL to 4 mg/mL (Figure 5). The results demonstrate that the cell vitality of the sample group remains unaffected across different concentrations of JPHT-2, indicating that JPHT-2 is safe and non-cytotoxic. In the food-processing industry, antioxidants are crucial to inhibit food oxidation, with phenolic antioxidants such as BHA, BHT, and TBHQ being the most commonly utilized [9]. However, the current study suggests that synthetic antioxidants may have harmful effects. Overuse and excessive supplementation of phenolic antioxidants can lead to DNA damage, carcinogenicity, and apoptosis [39]. It harms the human body in two ways. The first is its own cytotoxicity. For example, BHA can induce concentration-dependent apoptosis of rat hepatocytes in the concentration range of 300–500 μM [39]. Secondly, the metabolites of phenolic antioxidants in the human body, such as TQ, BHT-QM, and BHTOH-QM, can induce oxidative stress and cause DNA damage, which is closely related to the formation of cancer [39,40]. In contrast, JPHT-2 demonstrates biological activity that is both healthy and safe compared to chemically synthesized antioxidants with detrimental health effects [9].

**Figure 6:**
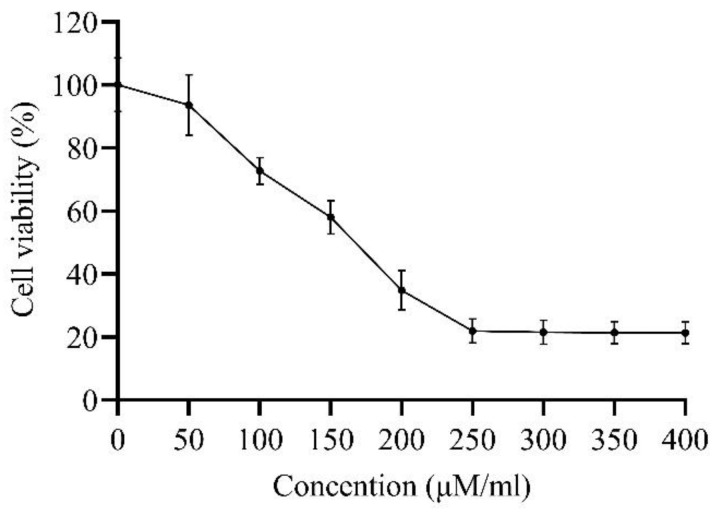
Survival rate of HaCaT treated with different concentrations of H_2_O_2_ (0–400 μM). Data are presented as means ± SD.

Hydrogen peroxide (H_2_O_2_) is a significant member of reactive oxygen species that can cause cellular damage and reduce the cell survival rate by reacting with ferrous ions in the cell to generate radicals [2,22]. H_2_O_2_ is relatively stable and easy to obtain, making it a commonly used substance for inducing oxidative damage in cell models. Cell modeling is a fast and cost-effective method, and its use in studying oxidative damage and the protective effects of antioxidants has been demonstrated in previous studies [2,22,41,42]. In particular, the HaCaT oxidative damage model is reliable for evaluating the antioxidant capacity of peptides [22]. Compared to animal models, cell models are simpler, more stable, and less prone to interference, making experimental results more reliable [41]. Figure 6 shows a decrease in the cell survival rate with increasing H_2_O_2_ concentration, with a cell survival rate of 58.03% at a concentration of 150 μM selected as the H_2_O_2_ treatment concentration for inducing oxidative damage.

### 3.5. Protective Effects on Oxidative Damage of HaCaT Cells

**Figure 7:**
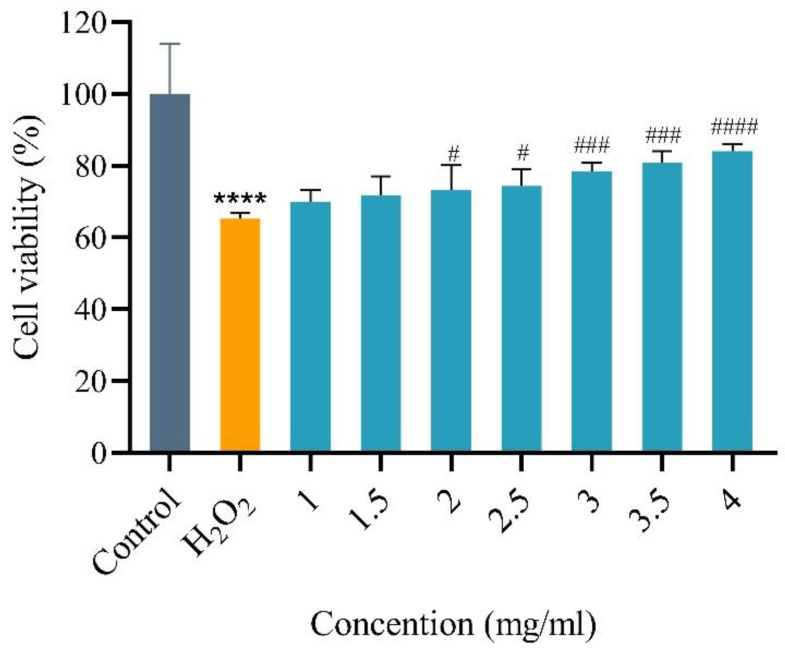
Cellular antioxidant activity treated with different concentrations of JPHT-2(1–4 mg/mL). **** *p* < 0.0001 vs. Control (to culture without treating with H_2_O_2_ and JPHT-2). # *p* < 0.05, ### *p* < 0.001, and #### *p* < 0.0001 vs. H_2_O_2_ (to culture treated only with H_2_O_2_). Data are presented as means ± SD.

JPHT-2 was added to the medium to demonstrate the antioxidant activity of jellyfish protein peptides at the cellular level. As shown in Figure 7, by comparing the results of the sample group and the H_2_O_2_ treatment group, we can find that HaCaT cells incubated with JPHT-2 (1–4 mg/mL) for 24 h could effectively resist the damage of H_2_O_2_ on HaCaT cells and significantly improved the survival rate.

**Figure 8:**
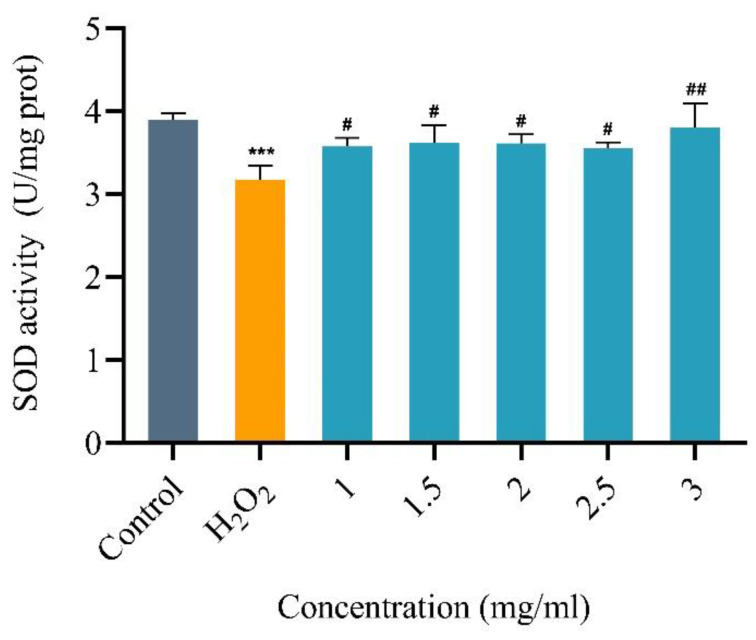
Cellular antioxidant activity treated with different concentrations of JPHT-2 (1–3 mg/mL). *** *p* < 0.001 vs. Control (to culture without treating with H_2_O_2_ and JPHT-2). # *p* < 0.05 and ## *p* < 0.01 vs. H_2_O_2_ (to culture treated only with H_2_O_2_). Data are presented as means ± SD.

Superoxide dismutase (SOD) is an important enzyme that plays a critical role in delaying aging, regulating metabolic capacity, and improving the immune function of the human body. It is widely found in animals, plants, microorganisms, and cultured cells, and can catalyze superoxide anion dismutation. Figure 8 shows the SOD activity in the oxidative damage treatment and sample groups. The results show that the SOD activity in the H_2_O_2_ treatment group was significantly reduced compared to the blank group, indicating that the oxidative damage model was successful. H_2_O_2_ can effectively damage HaCaT cells by oxidation. However, the incubation of HaCaT cells with JPHT-2 (1–3 mg/mL) for 24 h significantly reduced the damage of hydrogen peroxide on HaCaT cells, as evidenced by the higher SOD activity in the sample group compared to the oxidative damage group. These findings suggest that JPHT-2 may have a protective effect against oxidative-stress-induced damage at the molecular level.

### 3.6. Purification of Antioxidative Peptides

**Figure 9:**
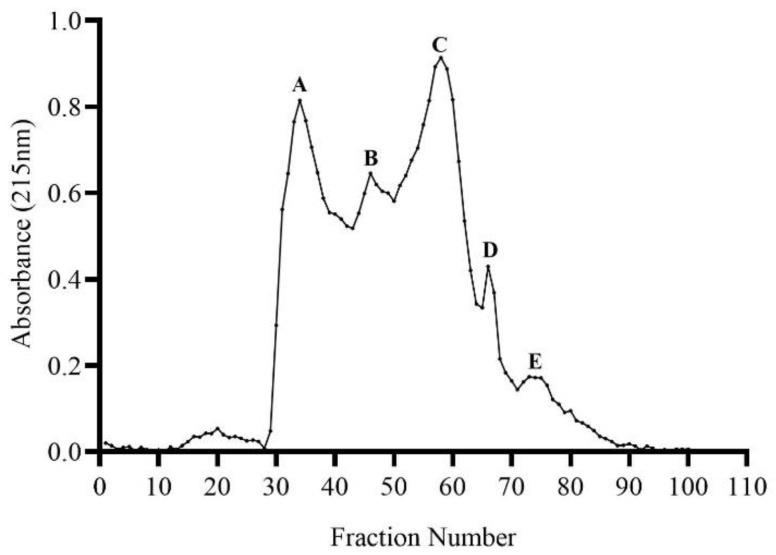
Elution profile of JPHT-2 separated using a Sephadex G-25. Fractions A–E were collected and prepared to be lyophilized powder.

**Figure 10:**
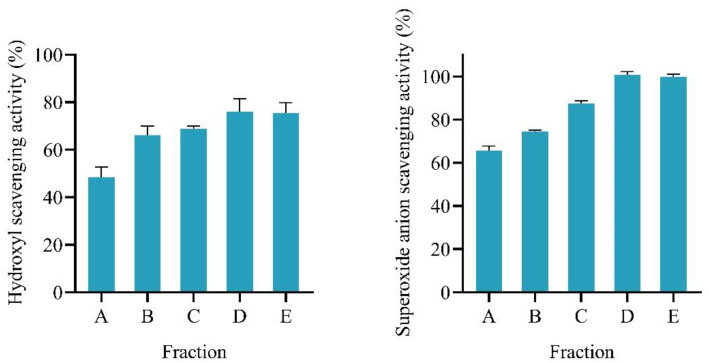
(**A**–**E**) show the hydroxyl radical and superoxide anion scavenging activities of different separated fractions (0.5 mg/mL) are presented as means ± SD.

Sephadex-G25 was used to separate JPHT-2. Five isolated components were obtained, as shown in Figure 9. The resulting components were collected and lyophilized, with the molecular weight decreasing from component A–E, and the molecular weight of component B–E are less than 1000 Da. We evaluated the hydroxyl radical and superoxide-anion-scavenging activities of fractions A-E and found that the antioxidant activity increased as the molecular weight decreased. Notably, in Figure 10, fractions D and E exhibited a significantly higher hydroxyl radical scavenging activity compared to JPHT-2, with superoxide anion activity 40% higher than that of JPHT-2. This suggests that peptides with a molecular weight of less than 1000 Da in JPHT-2 possess a stronger antioxidant activity, which is consistent with the finding that most antioxidant peptides comprise 2–20 amino acids and have a molecular weight of less than 3000 Da [24].

## 4. Discussion

Cnidarians, a diverse group of animals including jellyfish, sea anemones, and coral polyps, are a valuable source of bioactive substances such as terpenoids, phenolic derivatives, proteins, and peptides [43]. These compounds exhibit a wide range of biological activities, including antimicrobial, anti-inflammatory, and antitumor properties [44]. In particular, jellyfish are noteworthy due to their abundant population, ease of acquisition, and high protein content, which make them an attractive source of bioactive compounds. The production of bioactive peptides from jellyfish is a less complex and more productive process compared to other cnidarians, and they are widely distributed in oceans, making them easily harvestable. The discovery and development of new bioactive compounds from jellyfish have the potential to make significant contributions to various fields, including medicine, agriculture, and the food industry. Recent studies have indicated that small-molecular-weight peptides are more readily absorbed and utilized by the human body [7]. In this study, we successfully enhanced the hydrolysis effect of jellyfish by employing three enzymes, resulting in a major peptide fraction with a molecular weight of less than 1000 Da. The antioxidant activity of jellyfish peptides serves as a foundation for their potential efficacy in other biological activities of natural bioactive substances. We demonstrated the active oxygen-scavenging ability of these jellyfish peptides using various methods and further confirmed their protective effect against oxidative damage at the cellular level. According to the above findings, jellyfish have the potential to serve as a source of antioxidant-rich food. The utilization of multi-enzyme may offer a more effective means of utilizing jellyfish proteins.

Organic antioxidant peptides sourced from organisms are widely used in various industries, including cosmetics, food, and healthcare. Marine peptides possess several unique characteristics such as weak antigenicity, good biological compatibility, and low religious barriers, which set them apart from their terrestrial counterparts. Marine organisms such as fish and shellfish have already been processed to produce peptides with antioxidant properties, but their high economic value presents a challenge. Jellyfish are underutilized marine ecological disaster organisms due to their frequent blooms. Preparation of natural antioxidant peptides from jellyfish provides a new way to deal with the disaster organisms. Unlike chemically synthesized antioxidants such as BHA, BHT, and TBHQ, which have been linked to adverse health effects such as DNA damage, carcinogenicity, and apoptosis, jellyfish peptides exhibit favorable and secure biological activity [39]. It is a significant advantage of jellyfish peptides that they offer an alternative to synthesized antioxidants and can be utilized in a variety of applications. The results not only provide a reference for the application of jellyfish peptides in preventing various degenerative diseases related to oxidative damage, but also provide a theoretical basis for the utilization of jellyfish resources. However, its antioxidant mechanism, structure, and amino acid sequence are unknown. Future studies are needed to reveal the relationship between structure and activity.

## Figures and Tables

**Table 1:** Parameters for hydrolysis.

Types of Enzymes	pH	Temperature (°C)	Time (h)	Activity (U/g)
Trypsin	7.5	37	4	2.5 × 10^5^
Papain	6.5	55	4	8 × 10^5^
Pepsin	2.0	37	4	3 × 10^6^
Alcalase	9.0	50	4	2 × 10^5^
Flavourzyme	6.5	50	4	3 × 10^4^
Protamex	7.5	55	4	1 × 10^5^
Bromelain	6.5	55	4	6 × 10^5^

**Table 2:** Physical properties of jellyfish lyophilized powder.

Characters	Moisture (%)	Ash (%)
Rehydrated homogenate	95.54 ± 0.11	0.21 ± 0.02
Lyophilized powder	17.92 ± 0.38	4.47 ± 0.12

Results are obtained from means of three determinations ± standard deviation (SD).

**Table 3:** Orders of hydrolysis and abbreviations of hydrolysate.

Abbreviations of Hydrolysate	Orders of Hydrolysis		
	1	2	3
JPHD-1	Alcalase	Flavourzyme	
JPHD-2	Alcalase	Protamex	
JPHD-3	Flavourzyme	Alcalase	
JPHD-4	Flavourzyme	Protamex	
JPHD-5	Protamex	Alcalase	
JPHD-6	Protamex	Flavourzyme	
JPHT-1	Alcalase	Flavourzyme	Protamex
JPHT-2	Alcalase	Protamex	Flavourzyme
JPHT-3	Flavourzyme	Alcalase	Protamex
JPHT-4	Flavourzyme	Protamex	Alcalase
JPHT-5	Protamex	Alcalase	Flavourzyme
JPHT-6	Protamex	Flavourzyme	Alcalase

**Table 4:** Molecular weight distribution of JPHT-2.

Molecular Weight (Da)	Retention Time (min)	Relative Proportion (%)
>5000	<14.23	0.20
4000~5000	14.23~14.69	0
3000~4000	14.69~15.28	6.05
2000~3000	15.28~16.12	9.33
1000~2000	16.12~17.54	27.39
<1000	>17.54	57.01

**Table 5:** Regression equations and EC_50_ value of JPHT-2 and GSH.

Antioxidant Activity	JPHT-2			GSH
	Equation	R^2^	EC_50_ Value (mg/mL)	EC_50_ Value (mg/mL)
Hydroxyl	y = 25.66ln(x) + 58.454	0.9958	0.74	2.98
DPPH	y = 25.559ln(x) + 32.412	0.9584	1.99	0.06
Superoxide anion	y = 19.234ln(x) + 41.571	0.9929	1.55	1.15
Reducing power	y = 0.2225x + 0.0302	0.9933		

## Data Availability

Data is contained within the article.
